# Mitochondrial RNAs as Potential Biomarkers of Functional Impairment in Diabetic Kidney Disease

**DOI:** 10.3390/ijms23158198

**Published:** 2022-07-25

**Authors:** Stefania Di Mauro, Alessandra Scamporrino, Agnese Filippello, Maurizio Di Marco, Maria Teresa Di Martino, Francesca Scionti, Antonino Di Pino, Roberto Scicali, Roberta Malaguarnera, Francesco Purrello, Salvatore Piro

**Affiliations:** 1 Department of Clinical and Experimental Medicine, Internal Medicine, Garibaldi-Nesima Hospital, University of Catania, 95122 Catania, Italy; 8stefaniadimauro6@gmail.com (S.D.M.); alessandraska@hotmail.com (A.S.); agnese.filippello@gmail.com (A.F.); maurizio.dimarco@studium.unict.it (M.D.M.); antonino.dipino@unict.it (A.D.P.); robertoscicali@gmail.com (R.S.); salvatore.piro@unict.it (S.P.); 2 Department of Experimental and Clinical Medicine, Magna Graecia University, 88100 Catanzaro, Italy; teresadm@unicz.it; 3 Institute for Biomedical Research and Innovation (IRIB), National Research Council of Italy (CNR), 98164 Messina, Italy; francesca.scionti@irib.cnr.it; 4 Faculty of Medicine and Surgery, “Kore” University of Enna, 94100 Enna, Italy; roberta.malaguarnera@unikore.it

**Keywords:** circulating RNAs, biomarkers, DKD

## Abstract

Type 2 diabetes and renal damage are strictly linked. The progressive increase in T2D incidence has stimulated the interest in novel biomarkers to improve the diagnostic performance of the commonly utilized markers such as albuminuria and eGFR. Through microarray method, we analyzed the entire transcriptome expressed in 12 serum samples of diabetic patients, six without DKD and six with DKD; the downregulation of the most dysregulated transcripts was validated in a wider cohort of 69 patients by qPCRs. We identified a total of 33 downregulated transcripts. The downregulation of four mitochondrial messenger RNAs (MT-ATP6, MT-ATP8, MT-COX3, MT-ND1) and other two transcripts (seysnoy, skerdo) was validated in patients with eGFR stage G3 versus G2 and G1. The four messenger RNAs correlated with creatinine and eGFR stages, while seysnoy and skerdo were associated with white blood cell values. All transcripts correlated also with Blood Urea Nitrogen. The four mitochondrial messenger RNAs had a high diagnostic performance in G3 versus G2 discrimination, with AUC values above 0.8. The most performant transcript was MT-ATP6, with an AUC of 0.846; sensitivity = 90%, specificity = 76%, *p*-value = 7.8 × 10^−5^. This study led to the identification of a specific molecular signature of DKD, proposing the dosage of RNAs, especially mitochondrial RNAs, as noninvasive biomarkers of diabetes complication.

## 1. Introduction

Diabetic Kidney Disease (DKD) is a microvascular complication of diabetes, which occurs in 20–40% of patients [1]. It is the leading cause of end-stage renal disease (ESRD) in developed countries [2].

The pathogenesis of DKD is quite complex and not completely understood. However, it is possible to consider different pathways such as metabolic, hemodynamic, inflammatory, involved in DKD, with a composite interplay [3]. Among these, pivotal roles are played by oxidative stress and fibrosis, which could be considered bridges between different mechanisms responsible for renal damage in diabetic patients [4,5,6,7,8].

Even though advances have been made over the past few years in diagnosing and treating DKD patients, we are still not able to significantly reduce mortality among these patients. Interventions to slow down the progression of DKD, indeed, must start in the early stages of the disease. However, a big obstacle in clinical practice is the lack of biomarkers that can accurately identify diabetic patients who are at early risk of developing DKD and predict the progression of the condition [9]. 

To date, albuminuria and estimated glomerular filtration rate (eGFR) are the most common biomarkers used in clinical practice for DKD. However, these parameters show several limitations. Not all patients with DKD and reduced eGFR have increased albuminuria and it is possible to find increased albuminuria for different reasons, such as urinary tract infections, high blood pressure, high-protein diet, exercise, and fever [9,10]. Furthermore, albuminuria does not have a prognostic value. Microalbuminuria does not always progress to macroalbuminuria, but it can regress to a normoalbuminuric state [11]. As shown for albuminuria, eGFR has several limitations. For instance, P30 (a performance measure that represents the likelihood that the eGFR is within ±30% of the measured GFR) for the most common estimating equations is generally between 80 and 90% [2]. In addition, these equations are less precise for higher GFRs, and they are further limited by [12] variation in creatinine production because of age, gender, race, and body composition. In addition, at the beginning of the disease, this biomarker does not reflect the severity of the disease and when eGFR reaches the threshold of 60 mL/min/1.73 m^2^ almost 60% of the nephrons are already lost. Thus, it is a late biomarker of renal dysfunction and injury [13].

Therefore, it is clear that there is a need for a novel well-validated group of biomarkers to use in combination with conventional ones to improve the understanding of DKD pathophysiology and stratify patients according to their disease stage to conduct a tailored treatment [3]. DKD is a complication with multifaceted pathogenesis, therefore it is possible to consider biomarkers related to the different mechanisms involved in kidney injury, such as oxidative stress (i.e., 8-hydroxyseoxy-guanosine—8-OHdG), glomerular (i.e., nephrin) and tubular damage (i.e., urinary neutrophil gelatinase-associated lipocalin—NGAL—and urinary kidney injury molecule 1—KIM-1), inflammation (i.e., interleukin-6—IL-6), and fibrosis (i.e., type IV collagen) [3,14,15,16]. Unfortunately, none of these biomarkers are available in clinical practice. 

During the last few years, many studies have focused on the so-called ”omics-based biomarkers” of DKD. Indeed, transcriptomics, metabolomics and proteomic approaches have been used for urine or serum analysis of DKD patients [17]. As far as transcriptomics studies are concerned, they limited their analysis to miRNome expression profiles, although other classes of non-coding RNAs such as mRNAs and non-coding RNAs could be novel biomarkers for DKD [17]. These molecules, as well as microRNAs, are easily detectable and highly stable in biological fluids and against several pre-analytic conditions, for instance, repeated freeze-thaw cycles, long Room Temperature (RT) incubation and postponed processing of samples [18].

Moreover, there is evidence of long non-coding lncRNAs involved in DKD pathogenesis, such as Plasmacytoma Variant Translocation 1 (PVT1), ENSMUST00000147869, and CYP4B1-PS1-001 [19,20,21]. 

In agreement with these considerations, this study aimed to analyze the entire transcriptome in patients affected by DKD, in order to identify molecular fingerprints useful for the identification of new biomarkers of the pathology.

## 2. Results

### 2.1. DKD Is Characterized by a Specific Serum RNA Molecule Signature

**Figure 1:**
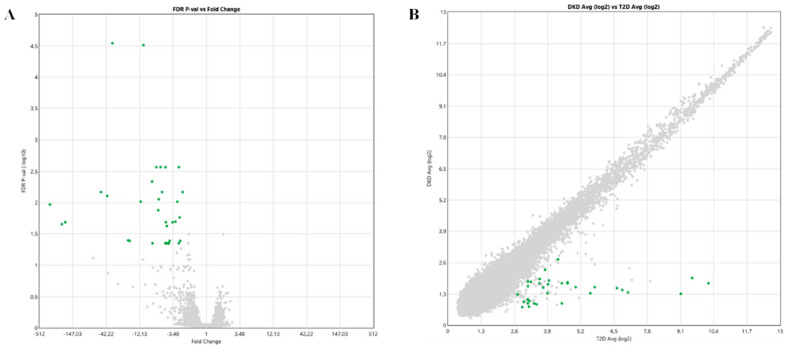
Volcano (**A**) and scatter plots (**B**) assessing the variation between diabetic patients with or without Diabetic Kidney Disease (DKD). The volcano plot visualizes the base 10 negative logarithm of the FDR-corrected *p*-values (Y-axis) and fold-change deregulation values (X-axis) (**A**). In the scatter plot, the values plotted on the X and Y axis are the averaged normalized signal values in each group (log_2_ scaled) (**B**). In both panels, green points indicate > 2.0-fold down-regulation of expression and grey points indicate < 2.0-fold-change in expression. FDR-corrected *p*-value < 0.05.

In order to identify a novel potential biomarker signature of DKD, the whole transcriptome expressed in the serum samples of 12 diabetic patients in the presence or absence of DKD (n = 6 ACR < 30 mg/g, eGFR ≥ 90 mL/min/1.73 m^2^ stage G1; n = 6 ACR between 30 and 299 mg/g, eGFR between 30 and 59 mL/min/1.73 m^2^ stage G3) was analyzed. Statistically significant dysregulated transcripts are represented as volcano and scatter plots in Figure 1 (panels A and B respectively). We identified a total of 33 dysregulated transcripts, all downregulated (FC ≤ 2, FDR *p*-value < 0.05), in patients with DKD with respect to diabetic patients without DKD. Supplementary Table S1 reports probe IDs, Gene symbols, fold-changes, FDR *p*-values and descriptions of dysregulated transcripts identified through microarray analysis.

### 2.2. Transcriptome Data Are Associated with Pathways of DKD Pathogenesis

**Figure 2:**
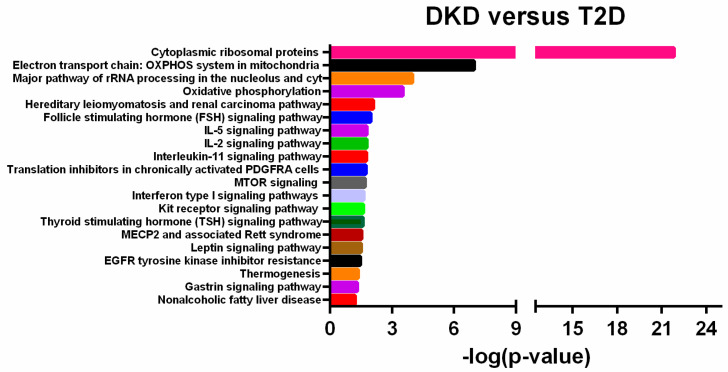
Enrichment pathway analysis: statistically significant pathways associated with Differentially Expressed (DE) transcripts in Diabetic Kidney Disease (DKD) patients versus diabetic patients without DKD (T2D). Y-axis reports the specific names of associated pathways, X-axis reports Fisher’s Exact Test values (−Log *p*-value ≥ 1.13).

To analyze the biological role of microarray-identified DE transcripts, we performed an enrichment pathway analysis through the use of TAC software. DE transcripts were statistically significantly associated with molecular pathways consistent with DKD pathogenesis such as inflammatory pathways (i.e., IL-5 signaling pathway, IL-2 signaling pathway, IL-11 signaling pathway), mitochondrial function (i.e., electron transport chain OXPHOS system, oxidative phosphorylation), UPR stress (i.e., cytoplasmic ribosomal proteins, major pathways of rRNA processing and cytosol) and fibrosis (i.e., VEGFA-VEGFR signaling pathway, interferon type 1 signaling pathway) (Figure 2).

The pathways that had a stronger statistical significance (Fisher Exact Test *p*-value > 3.52) were involved in UPR stress and mitochondrial functions. We reported the specific dysregulated transcripts associated to these pathways in Supplementary Table S2, and we also highlighted these transcripts in the volcano plot in Supplementary Figure S1.

### 2.3. Selected Transcripts Have a Decreasing Expression Trend Related to eGFR Stages

To confirm microarray data, we quantified the expression level of selected transcripts through Real-Time PCR in a wider independent validation cohort of 69 diabetic patients. Clinical and demographic patient data are reported in Table 1.

**Figure 3:**
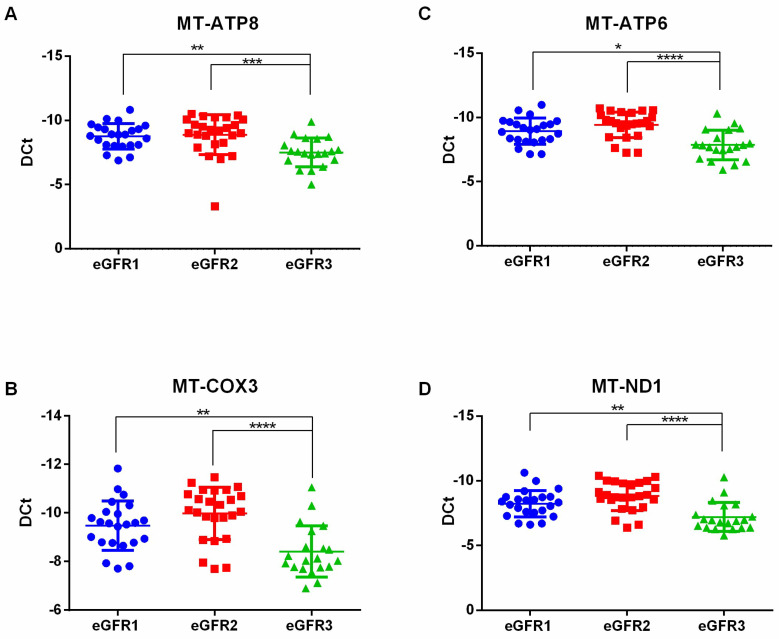
Dot plots of mitochondrial coding RNAs MT-ATP8 (panel **A**), MT-ATP6 (panel **B**), MT-COX3 (panel **C**) and MT-ND1 (panel **D**), validated through qPCR in serum samples of diabetic patients with increasing eGFR stages G1, G2 and G3. The Kruskal Wallis test was used for MT-ATP8 which presented a non-parametric distribution. The One-way ANOVA test was used for all the other transcripts showing parametric distributions. n = 69: G1 = 24, G2 = 25, G3 = 20. * *p*-value < 0.05 ** *p*-value < 0.01 *** *p*-value < 0.001 **** *p*-value < 0.0001.

**Figure 4:**
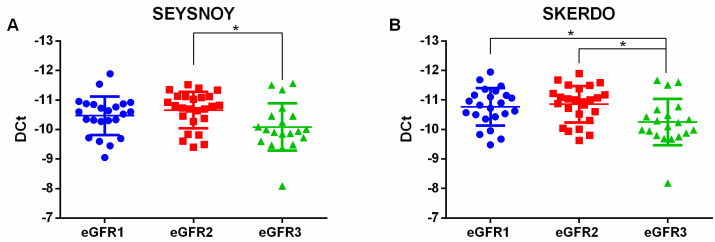
Dot plots of seysnoy (**A**) and skerdo (**B**) transcripts validated through qPCR in serum samples of diabetic patients with increasing eGFR stages G1, G2 and G3. The one-way ANOVA test was used for the two transcripts showing parametric distributions. n = 69: G1 = 24, G2 = 25, G3 = 20. * *p*-value < 0.05.

There was no significant difference between the studied groups, except for levels of uric acid, Blood Urea Nitrogen, creatinine, HCT systolic blood pressure, diastolic blood pressure, presence of hypertension condition, (One-way ANOVA or Kruskal Wallis test *p*-value < 0.05). We selected four coding RNAs, MT-ATP8, MT-ATP6, MT-COX3, and MT-MD1, and two “Aceview” database-reported transcripts seysnoy and skerdo. We selected these transcripts because, among the dysregulated transcripts (FDR *p*-value < 0.05), they had a strong FC dysregulation (FC < 18) and they presented higher levels of fluorescence signals in microarray data. Validation cohort patients have been grouped according to ACR (n = 36 normoalbuminuric subjects, n = 33 macroalbuminuric subjects) or according to eGFR stages (n = 24 G1, n = 25 G2, n = 20 G3). Taking into account ACR stratification, the dysregulation of any of the selected transcripts was confirmed. On the contrary, according to eGFR stratification, we validated the downregulation of all the 6 selected transcripts: four messenger RNAs and two “Aceview”-identified transcripts. Through Real-Time PCR we validated the downregulation of the four mRNAs, MT-ATP8, MT-ATP6, MT-COX3, and MT-ND1 and the skerdo transcript in G3 patients with respect to both G1 and G2 (Figure 3 and Figure 4). Seysnoy was under-expressed only in G3 patients versus the G2 comparison (Figure 4).

Table 2 reports *p*-values and fold-change values of analyzed transcripts in G3 versus G2 and G3 versus G1 comparisons. 

The downregulation of only the four messenger RNAs was also maintained in G3 versus G2 patients inside normoalbuminuria or microalbuminuria groups considered separately. The results of these latter comparisons are shown in Supplementary Table S3 and Figures S2 and S3. 

### 2.4. Validated Transcripts Are Associated with Patients’ Clinical Data

We performed a correlation analysis to test the hypothesis of a potential correlation between analyzed transcript expression values and patients’ clinical data. In agreement with Real-Time validation data, as expected, we found a correlation between all the transcripts and serum creatinine, as well as eGFR. For seysnoy and skerdo, we also found a correlation with White Blood Cell count (WBC). Blood urea nitrogen correlated with all analyzed transcripts. Since creatinine, eGFR, WBC and BUN had a nonparametric distribution, the correlation analysis between these clinical parameters and the expression levels of the validated transcripts was evaluated by the Spearman test. Table 3 shows the *p*-values of the correlation analysis.

### 2.5. ROC Curve Analysis

**Figure 5:**
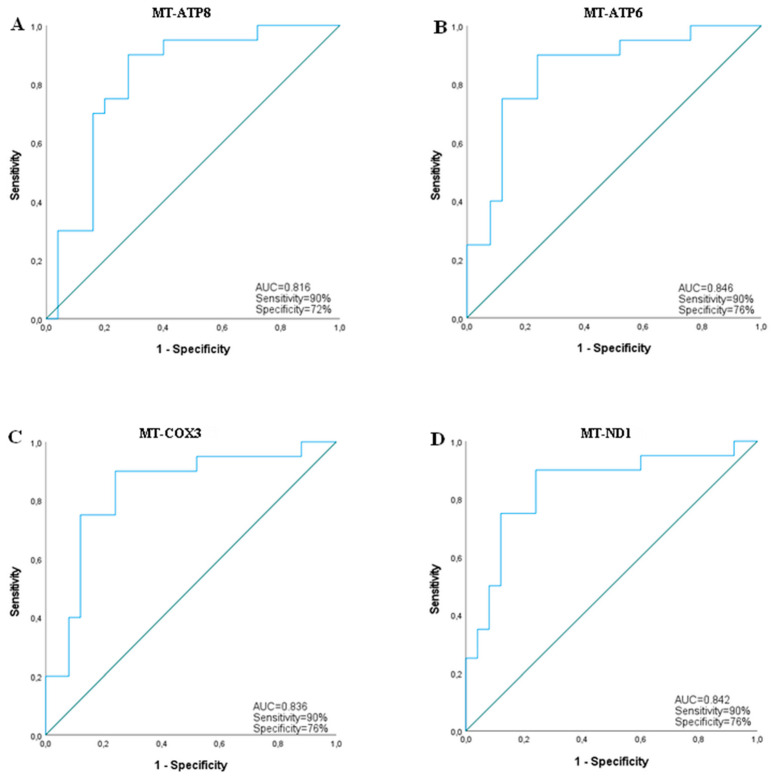
Receiver Operating Characteristic (ROC) analysis for predicting MT-ATP8 (**A**), MT-ATP6 (**B**), MT-COX3 (**C**) and MT-ND1 (**D**) as biomarkers of kidney function impairment in DKD (eGFR stage G3 versus G2).

**Figure 6:**
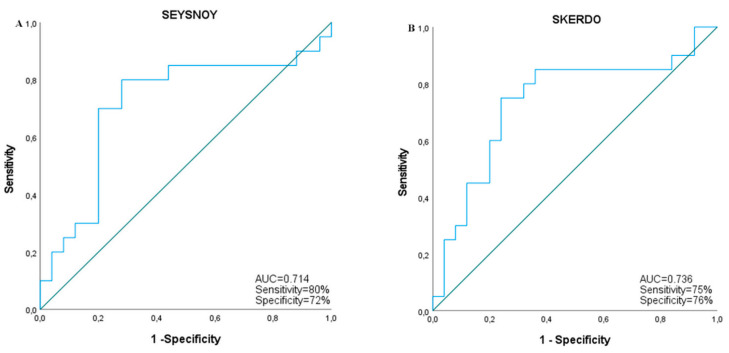
Receiver Operating Characteristic (ROC) analysis for predicting seysnoy (**A**) and skerdo (**B**) as biomarkers of kidney function impairment in DKD (eGFR stage G3 versus G2).

Performing ROC curve analysis, we analyzed the diagnostic performance of validated transcripts for G3 versus G2 patient discrimination. All analyzed transcripts reached statistical significance in ROC curve analysis. The four messenger RNAs showed high diagnostic performances, with AUC values above 0.80. The two “Aceview”-identified transcripts had lower diagnostic performances, with AUC values above 0.7. Since patients were stratified according to eGFR, in our cohort this parameter had an AUC value equal to 1, while albuminuria ROC curve analysis did not reach the statistical significance (AUC = 0.445, CI = 0.275–0.615, *p*-value = 0.527). Table 4 reports the AUC value, confidence interval, sensitivity, specificity and *p*-value data of each transcript for G3 subject identification. Figure 5 and Figure 6 show ROC curves of messenger RNAs, and seysnoy and skerdo respectively. 

## 3. Discussion

T2D and renal damage are inextricably linked. The increase in the incidence of T2D and the clinical relevance of DKD, determining an important rise in cardiovascular morbidity and mortality [19], explain the scientific interest in this complication. Taking into account the clinical relevance of DKD, subjects at higher risk to develop this complication should be identified earlier in order to address the main efforts for prevention and treatments that may arrest and prevent further disease progression. Currently, the biopsy is the gold standard for kidney disease diagnosis, however, it is highly invasive and has a risk of complications. Most DKD patients do not undergo renal biopsy [20]. Albuminuria and estimated glomerular filtration rate (eGFR) are the most commonly used diagnostic/prognostic biomarkers in clinical practice. Since both markers have several limitations [13], the identification of novel sensitive and specific biomarkers represents a pivotal clinical challenge. During recent years, circulating non-coding RNAs have received increasing interest. A lot of studies analyzed microRNA expression in several specimens, including serum and urine, in an attempt to identify novel diagnostic/prognostic biomarkers [21,22]. However, neither of these microRNAs have been introduced in clinical practice. 

This study supports the idea that other classes of RNA molecules could represent useful biomarkers in DKD. Indeed, similarly to microRNAs [23], other RNA transcripts are resistant to endogenous RNase degradation and easily detectable in biological fluids through basic molecular biology techniques [18]. Only a few studies analyzed the detection of messenger RNAs or ncRNA for DKD diagnosis/prognosis. Messenger RNAs have been mainly detected in urine sediment specimens. Song-Tao Feng et al. reported that the mRNAs coding for chemokines, more specifically CCL5 and CXCL1 mRNA levels, were upregulated in the urinary sediment of patients with 91 DN versus 60 controls, and were negatively correlated with eGFR [24]. Gang Wang demonstrated that in urine sediment samples, nephrin levels are significantly higher, and WT-1 levels were significantly lower in 21 diabetic patients than in 9 controls. However, this study failed in determining a correlation between these podocyte-related biomarkers and kidney function decline as estimated through eGFR [25]. Similarly, Min Zheng et al. reported that the podocyte-associated biomarkers podocalyxin, CD2-AP, α-actin4, and podocin were upregulated in 51 diabetic patients with DKD versus 13 controls. All target molecules were significantly correlated with urinary albumin, and only podocalyxin was inversely associated with eGFR [26]. As far as ncRNAs are concerned, few circulating lncRNAs have been reported to be dysregulated in serum samples of DKD patients. For instance, Chun Zhao reported that PANDAR is upregulated in diabetic patients and higher in DKD patients with massive proteinuria than microalbuminuric patients and negatively correlated with eGFR [27]. In another study, Jujie Gao et al. reported that NR_033515 was significantly increased in the serum of DKD patients and was related to the different stages of DKD. In addition, this noncoding RNA was also positively associated with diagnostic markers of DKD (KIM-1 and NGAL) [28]. 

We used a high-throughput approach for the analysis of the whole transcriptome expressed in serum samples in order to identify “molecular fingerprints”, useful for a more accurate definition of DKD diagnosis and prognosis. Through this approach, we identified 33 downregulated transcripts (FDR-corrected *p*-value < 0.05) in the serum of DKD patients in comparison with T2D patients without DKD (n = 12: 6 CKD, 6 T2D). Interestingly, the computational analysis showed that these transcripts were associated with pathways consistent with DKD pathogenesis, including inflammatory pathways, mitochondrial dysfunction, UPR stress and fibrosis. After that, we selected six of the most dysregulated transcripts (four coding RNAs: MT-ATP8, MT-ATP6, MT-COX3, MT-ND1 and: seysnoy and skerdo according to the Aceview database) and we validated their downregulation in a wider independent cohort of diabetic patients grouped according to ACR or eGFR stages. Identified transcripts were progressively downregulated according to eGFR stages with a strong statistical significance (n = 24 G1, n = 25 G2, n = 20 G3). In agreement with this data, we found a correlation between all the transcripts and serum creatinine, BUN and eGFR. Thus, identified biomarkers correlated with progressive functional kidney decline in the context of DKD. Furthermore, messenger RNAs had considerable diagnostic power for the discrimination of G3 versus G2 patients with AUC values above 0.8. The most performant transcript was ATP6 with AUC values of 0.846 (*p*-value = 7.8 × 10^−5^, sensitivity: 90%, specificity: 76%). Multivariate logistic analysis, performed by combining the expression data of all the identified transcripts and each possible combination of them, did not improve the identified biomarker diagnostic performance. This could be due to the problematic amount of collinearity in transcript expression data. Indeed the variance inflation factor (VIF) of analysed transcripts was above 5 and 10. In this condition, the redundance of data does not allow an improvement of the diagnostic performance in multivariate logistic analysis or potential combinatorial indices.

It is important to highlight that the downregulation of coding RNAs was also maintained in G3 versus G2 comparisons inside normoalbuminuria or microalbuminuria groups considered separately. This is particularly relevant for normoalbuminuric patients because a substantial proportion of T2D patients with DKD are normoalbuminuric and do not develop microalbuminuria (non-albuminuric phenotype). Hence, eGFR is the only available biomarker in this subgroup [13].

Interestingly, the four validated coding RNAs are subunits of the mitochondrial respiratory chain encoded by the mitochondrial genome. MT-ATP8 and MT-ATP6 respectively encode for the Fo subunits eight and six of the mitochondrial ATP synthase (complex V). MT-CO3 encodes for the subunit three of cytochrome c oxidase (complex III), and MT-ND1 for the subunit one of NADH: Ubiquinone oxidoreductase (complex I). 

It is important to highlight that blasting the sequences of seysnoy and skerdo through BLAT of UCSC browser, these two transcripts actually represent two different regions of the same mithocondrial transcript, MT-RNR1 mitochondrially encoded 12S ribosomalRNA. As is widely known, mitochondrial 12S rRNA is a constituent of mitoribosomes, the function of which are essential for the biogenesis of the oxidative phosphorylation system and for mitochondrial function in general [29]. Interestingly, Wuping Yang, et al. reported that the lncRNA NF582-AS1 overexpression induces a decrease of MT-RNR1 expression, followed by the inhibition of MT-CO2 (mitochondrially encoded cytochrome c oxidase II). On the contrary, MT-RNR1 overexpression reversed the decreased MT-CO2 expression [30].

It is widely known that mitochondrial dysfunction plays a pivotal role in DKD pathogenesis. It has been demonstrated that the assembling of Electron Transport Chain ETC complexes and supercomplexes is impaired in diabetes [31,32]. As far as DKD is concerned, several studies reported that ETC subunits are downregulated. For instance, through immunostaining experiments it has been demonstrated that cytochrome c oxidase (complex IV) is reduced in the kidneys of patients with diabetic nephropathy versus control subjects [33]; furthermore, it has been reported that the complex IV subunit MT-CO2 is lower in human postmortem glomeruli of T2D patients with eGFR ≤ 30 mL/min/1.73 m^2^, with respect to patients with higher eGFR or controls [34]. The alteration of ETC complex formation and the downregulation of complex subunits are consistent with the reduction of ETC functional activity. Several studies, indeed, reported that complex I, III, and/or IV activities are progressively reduced as DKD progresses in animal models [35]. In agreement with this data, we can hypothesize that the downregulation of RNA molecules coding for specific subunits of ETC in serum samples of patients with progressive decreasing eGFR values mirrors the ETC impairment/downregulation that occurs at the histological level in DKD. 

Since mitochondrial dysfunction is central to DKD pathogenesis, few other studies suggested the dosage of specific mitochondrial molecules/metabolites in blood or urine for DKD diagnosis and progression monitoring. In this context, the study conducted by Kumar Sharma et al. is very relevant. The authors, applying a metabolomics approach, identified 12 mitochondrial metabolites significantly reduced in diabetic patients with DKD versus patients without DKD; similarly to our results, this suggested a global suppression of mitochondrial activity in DKD. The same authors also demonstrated that urine exosome mitochondrial DNA (mtDNA) was reduced [33] in patients with DKD versus controls. Similarly, Ghada Al-Kafaji demonstrated that patients with DKD had lower mtDNA than patients with T2D and healthy controls. Furthermore, mtDNA had a decreasing expression trend, ranging from normoalbuminuric to microalbuminuric and macroalbuminuric groups (*p* < 0.01). mtDNA was also directly correlated with eGFR [36]. Only one study evaluated mtDNA levels in a longitudinal cohort of 19 patients with a follow-up of 24 months in supernatant, urinary sediment, or at the intra-renal level. Through univariate Cox regression analysis, the authors reported that there was no significant relation between mtDNA levels and renal survival [37].

Our cross-sectional study represents a starting point for the identification of non-coding RNAs and mRNAs as biomarkers for DKD. However, these data should be confirmed in a larger independent external cohort. Furthermore, in order to establish if identified RNA molecules can have a prognostic value, their downregulation should be confirmed in longitudinal cohorts, analyzing transcript expression serially and in association with the variation of other functional parameters. Although eGFR represents the main reference biomarker in clinical practice, once further validated in longitudinal cohorts these data could provide novel additional biomarkers for DKD, especially for patients who develop DKD and remain in a normoalbuminuric stage. Even with these limitations, this study offers a new and promising approach to biomarker discovery for DKD. 

## 4. Materials and Methods

### 4.1. Study Population

Diabetic patients were enrolled by the Internal Medicine Unit of the Garibaldi-Nesima Hospital, University of Catania. 

Diabetes patients presented fasting blood glucose ≥126 mg/dL and HbA1c ≥ 6.5% (48 mmol/mol) [38]. In order to define the presence of DKD complications, the Albumin/Creatinine Ratio (ACR) and eGFR were estimated. Our study included a total of 81 patients. 

12 patients represented the discovery cohort: 6 diabetic patients without DKD: ACR < 30 mg/g (normoalbuminuria), eGFR ≥ 90 mL/min/1.73 m^2^ (stage G1); 6 diabetic patients with DKD: ACR between 30 and 299 mg/g (microalbuminuria); eGFR between 30 and 59 mL/min/1.73 m^2^ (stage G3).

69 patients were included in the validation cohort that was grouped according to ACR (n = 36 normoalbuminuric subjects, n= 33 microalbuminuric subjects) or eGFR (n = 23 stage G1: eGFR ≥ 90 mL/min/1.73 m^2^, stage G2: n = 25 eGFR between 60 and 89 mL/min/1.73 m^2^, n = 20 stage G3: eGFR between 30 and 59 mL/min/1.73 m^2^). 

We excluded from our study patients with type 1 diabetes mellitus, nondiabetic kidney disease, hepatic diseases, autoimmune disorders, cancer, and diabetes mellitus complicated with cardiovascular diseases. For all patients, clinical, biochemical, pharmacological, and anthropometric data were collected.

### 4.2. Sample Processing

Blood samples were maintained at room temperature for one hour and subsequently centrifuged at 3500 rpm at 4 °C for 15 min in order to allow serum separation from whole blood. Serum was further centrifuged in order to remove eventual cell debris. The upper-layer supernatant was collected, and aliquots were stored at −80 until analysis.

### 4.3. RNA Extraction

Total RNA extraction was performed from 400 μL of serum by using the miRNeasy mini kit (QIAGEN) according to the manufacturer’s instructions. RNA elution was performed in a final volume of 200 μL. RNA concentration and quality assessment was performed by using the NanoDrop One (Thermo Fisher Scientific) [38,39,40,41].

### 4.4. Microarray Analysis

The high-throughput profiling of coding/non-coding RNAs in serum samples of six diabetic patients with DKD, and six matched diabetic patients without DKD, was performed by using Clariom D Pico Assay (Thermo Fisher Scientific, Milan, Italy) technology according to the manufacturer’s instruction [42,43].

### 4.5. In Silico Analysis

In order to highlight a potential association between microarray-identified dysregulated transcripts and molecular pathways consistent with DKD pathogenesis, we performed enrichment pathways analysis by using Transcriptome Analysis Console 4. This retrieves canonical biological pathways from the WikiPathways (Thermo Fisher Scientific, Santa Clara, CA, USA) database and returns *p*-values applying a two-sided Fisher’s Exact Test (−log *p*-value ≥ 1.13) [42,43].

### 4.6. Validation by qPCRs

To validate microarray results, the expression of six candidate transcripts among the most dysregulated ones (4 mRNAs and 2 “Aceview” reported transcripts) were analyzed in the serum samples of the validation cohort (n = 69) through Real-Time PCR (Power SYBR Green RNA-to-CT1-Step Kit (Thermo Fisher Scientific)) [44,45], in a QuantStudio 5 system (Thermo Fisher Scientific). ACTB was used as reference gene and relative quantification data were determined through the 2^−ΔΔCt^ method. The sequences of primers used in qPCRs are reported in Table 5.

### 4.7. Statistical Analysis

Microarray raw data normalization and the identification of Differentially Expressed (DE) transcripts were performed through *Transcriptome* *Analysis Console* (TAC) *v 4.* software according to the following parameters: Analysis Type: Expression Gene, Summarization Method: Gene Level—RMA, FDR *p*-Value < 0.05.

In order to check the normal distribution of clinical and expression data, three different normality tests were applied: the D’Agostino-Pearson omnibus test, the Shapiro–Wilk normality test and the Kolmogorov–Smirnov normality test. The unpaired *t*-test or Mann–Whitney U-test was used to analyze the statistical significance of expression data for parametric or no parametric data in normoalbuminuric versus microalbuminuric patient comparison. The one-way ANOVA or Kruskal–Wallis test was used to analyze the statistical significance of expression results for normal or no normal data in patients with increasing stages of eGFR (stages 1, 2 and 3).

We performed linear regression analysis or Spearman correlation (respectively, for normally or not normally distributed data) in order to identify a potential relationship between transcript expression values and subject clinical data. The statistical analyses were performed by using GraphPad Prism 6.0 (GraphPad Software, Inc., San Diego, CA, USA).

ROC curves and AUC were used to assess the diagnostic performance of identified differentially expressed RNAs for discrimination of eGFR severity grades of DKD. SPSS PASW Statistics V.27 was used for ROC curve analysis [42,43].

## 5. Conclusions

This study proposes the dosage of coding and non-coding RNAs as biomarkers of DKD. An important finding of our work is that addressing the biomarker discovery in the expression analysis of mitochondrially encoded RNAs could pave the way to the identification of novel DKD biomarkers.

## Figures and Tables

**Table 1:** Demographic and clinical data of the validation cohort. WC: Waist Circumference; BUN: Blood Urea Nitrogen; HbA1c: Hemoglobin A1; WBC: White Blood Cells; HCT: Haematocrit; BP: Blood Pressure.

Clinical Parameters	G1	G2	G3	*p*-Value
Age	67.30 ± 4.79	68.00 ± 5.10	70.60 ± 4.97	0.0825
Gender (% M)	78.26	80.77	75.00	0.8953
BMI (kg/m^2^)	27.25 (25.92–33.49)	29.17 (27.90–32.46)	29.76 (24.98–34.69)	0.6300
WC (cm)	104.10 ± 14.15	108.60 ± 12.97	107.50 ± 9.43	0.4900
AST (UI/L)	24.00 (22–29)	25.00 (21–34.50)	24.00 (21.50–30)	0.8400
ALT (UI/L)	31.00 ± (21–38)	25.50 ± (19–45)	18.50 ± (13–29.50)	0.0600
COL. TOT (mg/dL)	158.00 (122–180)	181.50 (143.5–207)	171.50 (141.3–209.5)	0.1700
HDL (mg/dL)	47.96 ± 9.38	45.65 ± 12.78	47.25 ± 11.20	0.7700
LDL (mg/dL)	75.40 (51.60–100.60)	96.70 (78.25–126.70)	77.40 (68.25–139.30)	0.0900
Triglycerides (mg/dL)	146.00 (83–202)	126.00 (88.75–244)	133.50 (108–156)	0.9200
Uric acid (mg/dL)	5.17 ± 1.63	6.25 ± 1.51	6.59 ± 1.25	0.0100
BUN (mg/dL)	18.22 (21.50–26.64)	21.96 (23.71–28.97)	33.41 (38.43–48.13)	<0.0001
HBA1c (%)	7.70 ± 1.34	7.70 ± 1.64	8.00 ± 1.35	0.9400
Creatinine (mg/dL)	0.73 (0.66–0.81)	0.96 (0.85–1.05)	1.40 (1.08–1.48)	<0.0001
Albuminuria (mcg/mL)	25.00 (12–33)	24.50 (6.75–124.8)	17.00 (10.50–37.75)	0.7900
ALB/CREAT	26.00 (14–45)	32.00 (8.5–72.5)	28.50 (13.25–89)	0.7900
WBC (μL^−1^)	7900 (5700–8900)	6200 (5200–8125)	7550 (5800–8875)	0.1400
HCT (%)	44.40 (42.20–46)	42.60 (39.93–45.13)	40.05 (37.60–41.70)	0.0100
Systolic BP (mmHg)	120.7 ± 11.20	125 ± 10.54	129.8 ± 11	0.0277
Dyastolic BP (mmHG)	75 ± 7.43	79.36 ± 6.90	80.65 ± 6.30	0.0210
Hyperthesis subjects (% H)	58.3	68	95	0.0208

**Table 2:** *p*-values and fold-change expression values of selected transcripts analyzed through Real-Time PCR in the validation cohort in G3 versus G2 or G1 comparisons.

Transcripts	FC G3 vs. G1	FC G3 vs. G2	*p*-Value G3 vs. G1	*p*-Value G3 vs. G2
MT-ATP8	−2.6	−3.3	0.0070	0.0003
MT-ATP6	−2.4	−3.4	0.0300	<0.0001
MT-COX3	−2.8	−4.1	0.0040	<0.0001
MT-ND1	−2.6	−3.7	0.0080	<0.0001
Seysnoy	-	−1.8	0.1367	0.0200
Skerdo	−1.6	−1.8	0.0400	0.0100

**Table 3:** *p*-Values of Spearman correlation analysis between the expression levels of analyzed transcripts and clinical patients’ data.

Transcripts	Creatinine (mg/dL)	eGFR ckd-epi (mL/m × 1.73 m^2^)	WBC (μL^−1^)	BUN (mg/dL)
MT-ATP8	0.0023	0.0031	0.0602	0.0010
MT-ATP6	0.0112	0.0156	0.0889	0.0082
MT-COX3	0.0135	0.0155	0.1394	0.0050
MT-ND1	0.0101	0.0158	0.1168	0.0046
skerdo	0.0078	0.0063	0.0170	0.0252
seysnoy	0.0354	0.0303	0.0174	0.04403

**Table 4:** *p*-values and fold-change expression values of selected transcripts analyzed through Real-Time PCR in the validation cohort in G3 versus G2 or G1 comparisons.

Transcripts	AUC	CI	*p*-Value	Sensitivity	Specificity
MT-ATP8	0.816	0.687–0.945	2.0 × 10^−6^	90%	72%
MT-ATP6	0.846	0.728–0.964	7.8 × 10^−5^	90%	76%
MT-COX3	0.836	0.710–0.962	1.2 × 10^−4^	90%	76%
MT-ND1	0.842	0.718–0.966	9.4 × 10^−5^	90%	76%
seysnoy	0.714	0.580–0.892	1.0 × 10^−2^	75%	76%
Skerdo	0.736	0.580–0.892	3.0 × 10^−3^	80%	72%

**Table 5:** Primer sequences of transcripts analyzed through qPCRs.

Transcripts	Primer Sequences
MT-ATP8	F 5′ ACAGTGAAATGCCCCAACTAAAT 3′ R 5′ AGGGAGGTAGGTGGTAGTTTGTG 3′
MT-ATP6	F 5′ ACCTTCCCTCTACACTTATCATCTT3′ R 5′ CGTGCAGGTAGAGGCTTACT 3′
MT-COX3	F 5′ TTCACCATTTCCGACGGCAT 3′ R 5 GGCGGATGAAGCAGATAGTGA’ 3′
MT-ND1	F 5′ CGGGCTACTACAACCCTTCG 3′ R 5 AGATGTGGCGGGTTTTAGGG 3′
seysnoy	F 5′ TACCCCACTATGCTTAGCCCT 3′ R 5′ AGCTGTGGCTCGTAGTGTTC 3′
skerdo	F 5′ GGGTTGGTCAATTTCGTGCC 3′ R 5′ ACACTCTTTACGCCGGTTTCT 3′
ACTB2	F 5′ GAGCACAGAGCCTCGCCTTT 3′ R 5′GAGCGCGGCGATATCATCA 3′

## Data Availability

Not applicable.

## Supplementary Materials

The following supporting information can be downloaded at: https://www.mdpi.com/article/10.3390/ijms23158198/s1.
