# Supplementary material for: Mitochondrial RNAs as Potential Biomarkers of Functional Impairment in Diabetic Kidney Disease
Source: Int J Mol Sci. 2022 Jul 25;23(15):8198. doi: 10.3390/ijms23158198 (PMC9331991; doi:10.3390/ijms23158198)
Supplement: Supplementary file 1 [file ijms-23-08198-s001.zip › ijms-1806171-supplementary.pdf]

SUPPLEMENTARY MATERIALS

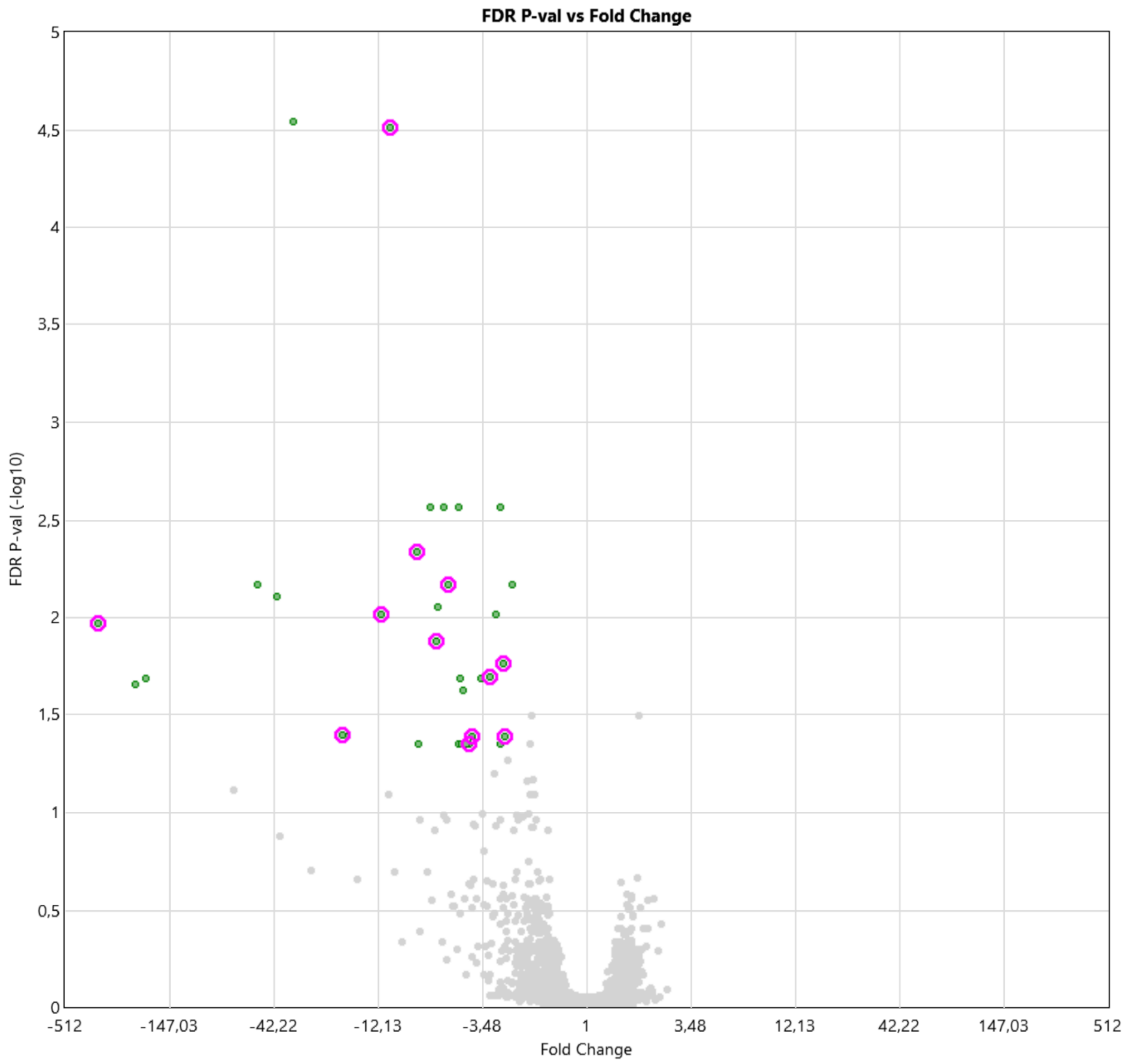

Supplementary Figure S1. Volcano plot assessing the variation between patients with or without Diabetic Kidney Disease (DKD). The volcano plot visualizes the base 10 negative logarithm of the FDR-corrected *p*-values (Y-axis) and fold change deregulation values (X-axis), green points indicate > 2.0-fold down-regulation of expression and grey points indicate < 2.0-fold change in expression. FDR-corrected *p*-value < 0.05. Dysregulated transcripts associated with UPR stress pathways or mitochondrial pathways are circled in pink.

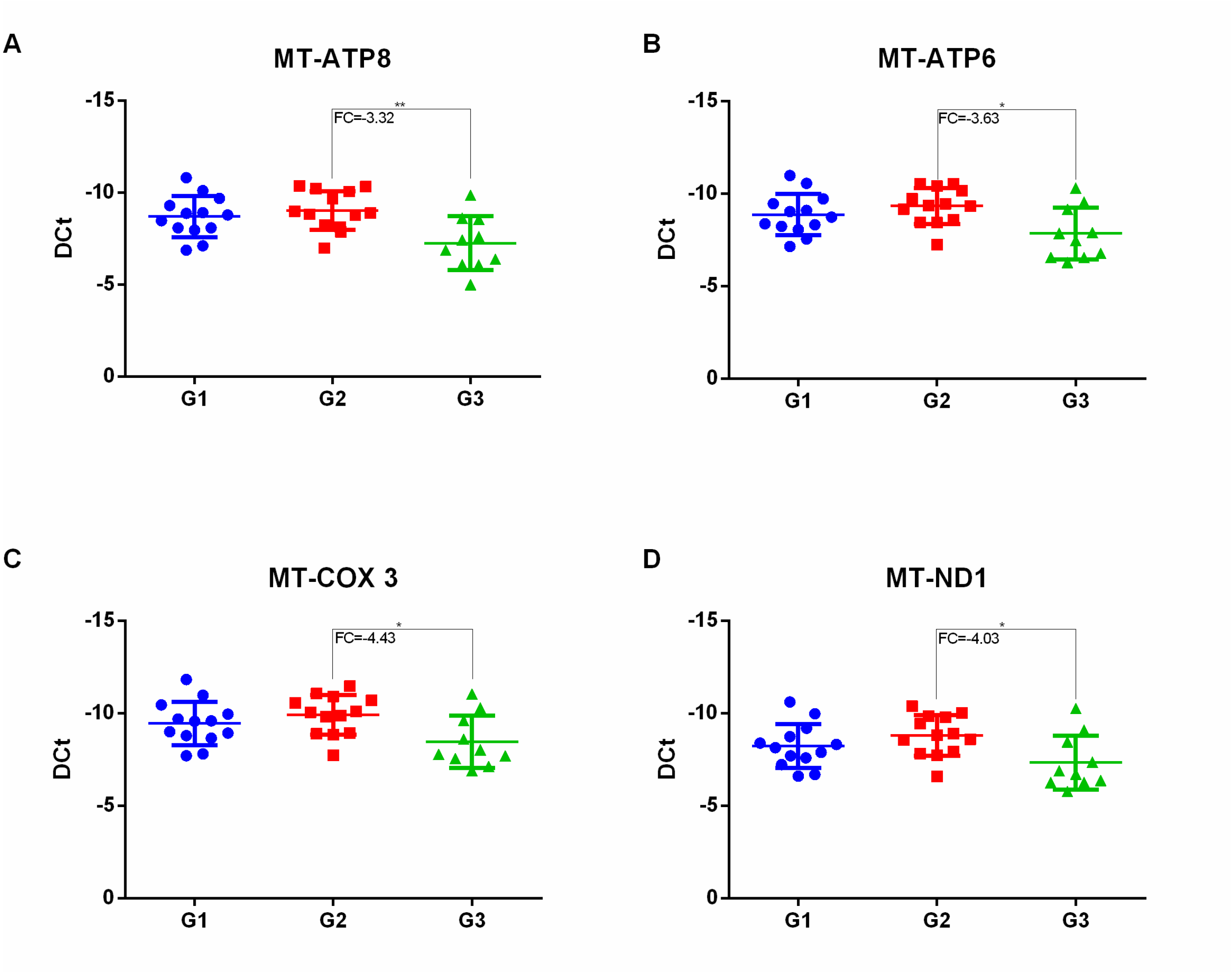

Supplementary Figure S2. Dot plots of mitochondrial coding RNAs MT-ATP8 (panel A), MT-ATP6 (panel B), MT-COX3 (panel C) and MT-ND1 (panel D) validated through qPCR in serum samples of normoalbuminuric diabetic patients with increasing eGFR stages G1, G2 and G3. The Kruskal Wallis test was used for all transcripts. n = 36: G1=13, G2=13, G3=10. *p-value <0.05 **p-value < 0.01

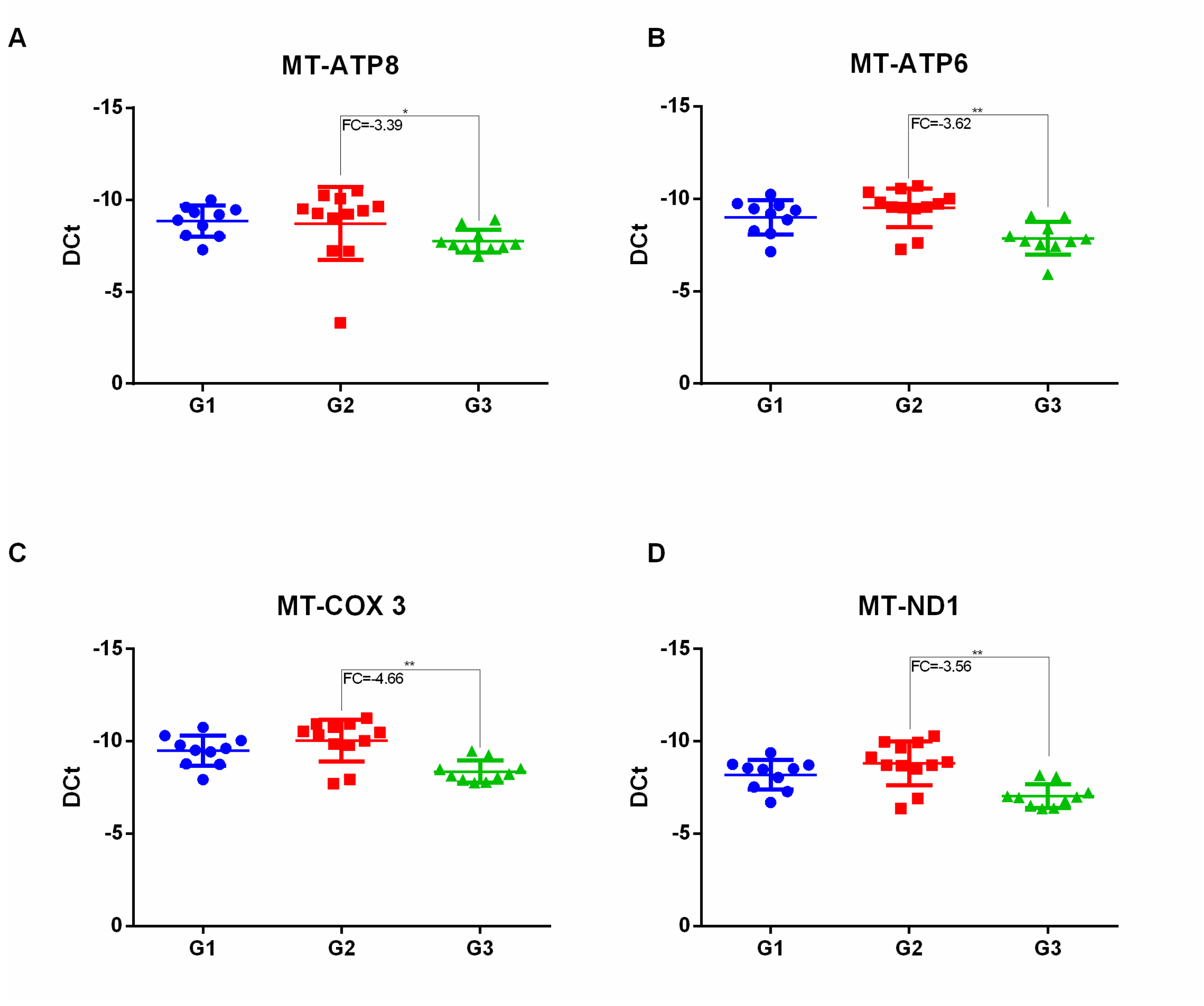

Supplementary Figure S3. Dot plots of mitochondrial coding RNAs MT-ATP8 (panel A), MT-ATP6 (panel B), MT-COX3 (panel C) and MT-ND1 (panel D) validated through qPCR in serum samples of microalbuminuric diabetic patients with increasing eGFR stages G1, G2 and G3. The Kruskal Wallis test was used for all transcripts. n = 33: G1=10, G2=13, G3=10. *p-value <0.05 **p-value <0.005.

Supplementary Table S1. List of dysregulated transcripts identified through microarray analysis, FC<2, FDR-corrected p-value < 0.05.

IDs	FC	FDR P-values	Gene Symbols	Descriptions
TC0X00009466.hg.1	-33,46	2,86E-05	RP5-1174J21.1	
TC0400008359.hg.1	-10,61	3,06E-05	RPL34	ribosomal protein L34
TC0700009597.hg.1	-6,55	0,0027	RNY3	RNA, Ro-associated Y3
TC0X00007546.hg.1	-5,5	0,0027	RPS23P8	ribosomal protein S23 pseudogene 8 [Source:HGNC Symbol;Acc:HGNC:35982]
TC1500007131.hg.1	-4,6	0,0027	Y_RNA	Y RNA [Source:RFAM;Acc:RF00019]
TC0100015884.hg.1	-2,82	0,0027	Y_RNA	Y RNA [Source:RFAM;Acc:RF00019]
TC0800010502.hg.1	-7,61	0,0046	RPS20; SNORD54	ribosomal protein S20; small nucleolar RNA, C/D box 54
TC0X00010755.hg.1	-51,23	0,0068	skerdo	Transcript Identified by AceView
TC0900009654.hg.1	-5,27	0,0068	RPS6	ribosomal protein S6
TC1100012226.hg.1	-2,44	0,0068	Y_RNA	Y RNA [Source:RFAM;Acc:RF00019]
TSUnmapped00000846.hg.1	-40,62	0,0078		
TC1200007387.hg.1	-5,89	0,0089	Y_RNA	Y RNA [Source:RFAM;Acc:RF00019]
TC2000008007.hg.1	-11,7	0,0097	RPS21	ribosomal protein S21
TC0700012340.hg.1	-2,96	0,0097		
TC0M00006432.hg.1	-346,47	0,0107	ND1	NADH dehydrogenase, subunit 1 (complex I)
TC0100010060.hg.1	-6,01	0,0133	RPS27	ribosomal protein S27
TC1100008612.hg.1	-2,69	0,0171	RPS28	ribosomal protein S28
TC0X00010648.hg.1	-3,17	0,0201	RPL39; SNORA69	ribosomal protein L39; small nucleolar RNA, H/ACA box 69
TC0300011792.hg.1	-194,01	0,0205	MTRNR2L2; MTRNR2L12	MT-RNR2-like 2; MT-RNR2-like 12
TC1600007712.hg.1	-4,54	0,0205	kleyweybu	Transcript Identified by AceView
TC0M00006433.hg.1	-3,55	0,0205	MT-TM	mitochondrially encoded tRNA methionine [Source:HGNC Symbol;Acc:HGNC:7492]
TC1300009757.hg.1	-221,72	0,0221	seysnoy	Transcript Identified by AceView
TC1300008309.hg.1	-4,42	0,0237	FTH1P7	ferritin, heavy polypeptide 1 pseudogene 7 [Source:HGNC Symbol;Acc:HGNC:3994]
TC0M00006439.hg.1	-18,76	0,0398	ATP8; ATP6; COX3	ATP synthase F0 subunit 8; ATP synthase F0 subunit 6; cytochrome c oxidase III
TC1400010741.hg.1	-3,98	0,0405	RPS29; RPL32P29	ribosomal protein S29; ribosomal protein L32 pseudogene 29
TC0400011114.hg.1	-17,74	0,0407	RP11-777B9.5	
TC0200016595.hg.1	-2,68	0,0407	RPL37A	ribosomal protein L37a
TC0700010444.hg.1	-4,6	0,0442	EEF1A1P6	eukaryotic translation elongation factor 1 alpha 1 pseudogene 6 [Source:HGNC

				Symbol;Acc:HGNC:3201]
TC1200010097.hg.1	-4,47	0,0442	leynar	Transcript Identified by AceView
TC2000008479.hg.1	-4,21	0,0442	Y_RNA	Y RNA [Source:RFAM;Acc:RF00019]
TC1700007383.hg.1	-4,1	0,0442	RPL23A; SNORD4B; SNORD42B;SNORD42A	ribosomal protein L23a; small nucleolar RNA, C/D box 4B; small nucleolar RNA, C/D box 42B; small nucleolar RNA, C/D box 42A
TC1700011363.hg.1	-2,8	0,0442	NACA2	nascent polypeptide-associated complex alpha subunit 2
TC1100010088.hg.1	-7,47	0,0445	ploglor	Transcript Identified by AceView

Supplementary Table S2. List of differential expressed transcripts involved in UPR stress pathways and mitochondrial pathways.

**UPR stress pathways**	**Dysregulated Transcripts**
Cytoplasmic ribosomal proteins	RPL23A, RPL34, RPL37A, RPL39, RPS6, RPS20, RPS21, RPS27, RPS28, RPS29
Major pathways of rrna processing and cytosol	RPS6, RPS27, RPS28
**Mithocondrial pathways**	**Dysregulated Transcripts**
Electron transport chain: oxphos system in mitochondria	MT-ND1, MT-COX3, MT-ATP6, MT-ATP8
Oxidative phosphorylation	MT-ND1, MT-ATP6

Supplementary Table S3. p-values and Fold Change expression values of selected transcripts analyzed through Real- Time PCR in the validation cohort in G3 versus G2 comparisons in normoalbuminuric (upper panel) or microalbuminuric state (lower panel).

NORMOALBUMINURIC STATE

	**FC G3 vs G2**	**p-value G3 vs G2**
**ATP8**	-3.32	**0.0092**
**ATP6**	-3.63	**0.0199**
**COX3**	-4.43	**0.0248**
**MT-ND1**	-4.03	**0.0292**
**SEYSNOY**	-	0.0926
**SKERDO**	-	0.0511

MICROALBUMINURIC STATE

	**FC G3 vs G2**	**p-value G3 vs G2**
**ATP8**	-3.39	**0.0363**
**ATP6**	-3.62	**0.0025**
**COX3**	-4.66	**0.0015**
**MT-ND1**	-3.56	**0.0013**
**SEYSNOY**	-	0.3064
**SKERDO**	-	0.3653
